# Colorectal Cancer Surgery Quality in Manitoba: A Population-Based Descriptive Analysis

**DOI:** 10.3390/curroncol28030206

**Published:** 2021-06-16

**Authors:** Iresha Ratnayake, Jason Park, Natalie Biswanger, Allison Feely, Grace Musto, Kathleen Decker

**Affiliations:** 1 Department of Epidemiology & Cancer Registry, CancerCare Manitoba, Winnipeg, MB R3E 0V9, Canada; afeely@cancercare.mb.ca (A.F.); gmusto@cancercare.mb.ca (G.M.); kdecker@cancercare.mb.ca (K.D.); 2 Department of Community Health Sciences, Rady Faculty of Health Sciences, University of Manitoba, Winnipeg, MB R3E 3P5, Canada; nbiswanger@cancercare.mb.ca; 3 Department of Surgery, Rady Faculty of Health Sciences, University of Manitoba, Winnipeg, MB R3E 3P5, Canada; JPARK@sbgh.mb.ca; 4 CancerCare Manitoba, Winnipeg, MB R3E 0V9, Canada; 5 Screening Programs, CancerCare Manitoba, Winnipeg, MB R3C 2B1, Canada; 6 Research Institute in Oncology & Hematology, CancerCare Manitoba, Winnipeg, MB R3E 0V9, Canada

**Keywords:** colorectal cancer, cancer surgery, surgical quality

## Abstract

Unwarranted clinical variation in healthcare impacts access, productivity, performance, and outcomes. A strategy proposed for reducing unwarranted clinical variation is to ensure that population-based data describing the current state of health care services are available to clinicians and healthcare decision-makers. The objective of this study was to measure variation in colorectal cancer surgical treatment patterns and surgical quality in Manitoba and identify areas for improvement. This descriptive study included individuals aged 20 years or older who were diagnosed with invasive cancer (adenocarcinoma) of the colon or rectum between 1 January 2010 and 31 December 2014. Laparoscopic surgery was higher in colon cancer (24.1%) compared to rectal cancer (13.6%). For colon cancer, the percentage of laparoscopic surgery ranged from 12.9% to 29.2%, with significant differences by regional health authority (RHA) of surgery. In 86.1% of colon cancers, ≥12 lymph nodes were removed. In Manitoba, the negative circumferential resection margin for rectal cancers was 96.9%, and ranged from 96.0% to 100.0% between RHAs. The median time between first colonoscopy and resection was 40 days for individuals with colon cancer. This study showed that high-quality colorectal cancer surgery is being conducted in Manitoba along with some variation and gaps in quality. As a result of this work, a formal structure for ongoing measuring and reporting surgical quality has been established in Manitoba. Quality improvement initiatives have been implemented based on these findings and periodic assessments of colorectal cancer surgery quality will continue.

## 1. Introduction

Variation in health care is an important yet complex issue [1,2]. Due to the complexity of health care systems, some unexplained variation is unavoidable [3]. However, it is “unwarranted clinical variation” that is of most concern. Unwarranted clinical variation is “variation that clearly isn’t explained by illness, medical science, or patient preference” [4] that impacts access, productivity, performance, and outcomes [3]. Studies have found that this variation exists at the local (between providers, hospitals, and geographic areas), national (between provinces and territories), and international (between countries) level [4,5,6]. Some of the underlying reasons for variation are related to the distribution of resources, clinical staff, training and skills, and healthcare funding.

A proposed strategy for reducing unwarranted clinical variation is to ensure that population-based information describing the current state of health care services is continuously available to clinicians and healthcare decision-makers [4]. The lack of these data results in stakeholders who may not clearly understand the current state of health care delivery. Decision-makers and clinical stakeholders need to collaborate to identify, measure, and implement quality improvement initiatives to reduce variation [3]. Significant collaboration efforts and strong clinical leadership are paramount to addressing variation in health care. Further to this, publicly sharing this information is key to engaging policymakers, patients, and the general public. While this is informally done in Manitoba (and other provinces) for cancer surgery, this project’s efforts formalized a structure for ongoing reporting and quality measurement. The objective of this study was to measure variation in colorectal cancer (CRC) surgical treatment patterns and surgical quality in Manitoba, and to identify areas for improvement.

## 2. Methods

### 2.1. Study Design and Data Sources

This study used a descriptive design to measure variation in colorectal cancer surgery. The data sources used were the Manitoba Cancer Registry (MCR), Hospital Discharge Abstracts Database (DAD), Medical Claims Database, Manitoba Health Insurance Registry (MHIR), and Statistics Canada 2006 Census. The MCR, DAD, and Medical Claims data were used to identify clinical information, and the MHIR and Statistics Canada data were used to determine some demographic and coverage information. A detailed description of how each data source was used has been previously published through an analogous project on breast cancer surgery [7].

### 2.2. Study Population

The study included Manitobans aged 20 years or older who were diagnosed with invasive cancer (adenocarcinoma) of the colon or rectum (International Classification of Diseases for Oncology (ICD-O) C18.0, C18.2-C18.9, C19.9, and C20.9) between 1 January 2010 and 31 December 2014. Non-adenocarcinoma pathology codes and cancers of the appendix were excluded. Manitoba is a Canadian province with publicly funded health care. The province is divided into five regional health authorities (RHAs) (Winnipeg Regional Health Authority, Prairie Mountain Health, Interlake-Eastern Regional Health Authority, Southern Health-Santé Sud, and Northern Regional Health Authority) which are responsible for health care delivery and governance.

Manitoba’s largest regional health authority is the Winnipeg Regional Health Authority, and it includes the city of Winnipeg (population 1518.8 per km^2^) and the northern town of Churchill (population density of 16.7 per km^2^). [8]. These two jurisdictions were merged into one RHA for administrative purposes. The overall population density for the WRHA is 1112.3 per km^2^. The city of Brandon is Manitoba’s second largest city (population density of 631.2 per km^2^) and part of the Prairie Mountain Health region (population density of 2.6 per km^2^). The Interlake-Eastern, Southern Health-Santé Sud, and Northern Regional Health authorities all have a population density <10 per km^2^ with most northern towns with <1 per km^2^. For example, in the Northern RHA a population of 74,000 people are spread out over 396,000 km^2^ resulting in a population density of 0.18 per km^2^ [8,9]. These geographical differences provide important contextual information that must be considered when assessing healthcare variation. This study will refer to the Winnipeg Regional Health Authority as the urban RHA and the other RHAs as rural RHAs 1 to 4.

### 2.3. Definition of Surgical Treatment

Colorectal cancer surgery was identified using the following Canadian Classification of Health Interventions (CCI) codes: 1.NK.76, 1.NM.76, 1.NM.77, 1.NK.87, 1.NM.87, 1.NM.89, 1.NM.91, 1.NQ.87, 1.NQ.89, 1.NK.87, and 1.NQ.90.

### 2.4. Outcomes

The selected process and quality outcome measures assessed were surgical approach, time between first colonoscopy and surgical resection for colon cancers, lymphadenectomy for colon cancers, and circumferential resection margin rate for rectal cancers. For this analysis, we wanted to focus on measures directly related to surgical processes that were less dependent on other factors on the cancer treatment pathway (such as administration and timing of adjuvant treatments). Surgical approach was defined as either open, laparoscopic, or per orifice, and was stratified by the RHA in which the surgery took place. The number of days between first colonoscopy and first resection was stratified by income quintile, the RHA in which the patient resided at diagnosis, and stage at diagnosis. Lymphadenectomy ≥12 nodes and circumferential resection margin (CRM), which are considered markers of quality indicators for colorectal cancer surgery [10,11], were stratified by RHA of residence and stage at diagnosis. Stage IV was excluded from the CRM indicator as the intention was to assess quality among surgeries with curative intent, and stage IV can contain both curative and palliative intent surgeries.

### 2.5. Statistical Analysis

The analyses comprised of calculating percentages and 95% confidence intervals using SAS version 9.4. All analyses were stratified by income quintile (Urban: U1 (lowest) to U5 (highest) and Rural: R1 (lowest) to R5 (highest)), RHA of surgery or residence at time of diagnosis (Winnipeg Regional Health Authority, Prairie Mountain Health, Interlake-Eastern Regional Health Authority, Southern Health-Santé Sud, or Northern Regional Health Authority), and stage at diagnosis (stages I to IV).

## 3. Results

During the 2010–2014 time period, 2459 individuals were diagnosed with colon cancer and 986 individuals were diagnosed with rectal cancer (Table 1). The median age of the cohort was 70 (interquartile range (IQR) = 79–60). Eighty percent of colon cancers and 69% of rectal cancers were diagnosed in individuals 60 years old and over. The distribution of colon cancers was similar among males and females, while a higher percentage of rectal cancers were diagnosed in males (63.8%). For colon and rectal cancers, most individuals were diagnosed at stages II and III (64.6% and 61.5%, respectively).

Among individuals diagnosed with colon cancer, 87.5% underwent a resection, 4.6% underwent a polypectomy, and 7.9% did not have a resection. Among those diagnosed with rectal cancer, 66.9% underwent a resection and 33.1% did not have a resection or a polypectomy. Open surgical approach was more common in both colon and rectal cancer surgeries (75.8% and 77.1%, respectively) (Table 2 and Table 3). Laparoscopic surgery was higher in colon cancer (24.1%) compared to 13.6% in rectal cancer. For colon cancer, the percentage of laparoscopic surgery by RHA ranged from 12.9% to 29.2%. Laparoscopic surgery for rectal cancer showed less variation, with a range of 13.2% to 18.1% by RHA. In addition to open and laparoscopic approaches, rectal cancer surgery also used a per orifice approach (9.2% of cases).

The median number of days between first colonoscopy and first resection was 40 days (Table 4) for individuals with colon cancer. Little variation was identified among urban income quintiles, however among rural income quintiles, the median ranged from 32 to 48 days. Individuals with stage IV cancer experienced the shortest median wait time (23 days for stage IV vs. 60 days for stage I).

An important quality indicator for colon cancer resection is removal of (lymphadenectomy) of ≥12 lymph nodes. In 86.1% of colon cancers, ≥12 lymph nodes were removed (Table 5). Little variation was identified between RHAs and all stages showed a high percentage of lymph node removal (74.8% to 90.9%). The negative circumferential resection margin rate for rectal cancers was 96.9%, ranging between 96.0% and 100.0% by RHA of residence.

## 4. Discussion

This study found variations in surgical treatment and quality for colorectal cancer in Manitoba between income quintiles, RHA, and stage of disease. Surgical approach is a potential indicator of surgical quality for colon cancers. The indications for laparoscopic surgery have expanded over time, and the impact on outcomes has been well researched [12]. Studies have demonstrated that laparoscopic surgery is a favourable alternative to open surgery for colon cancer, with superior short-term outcomes such as faster recovery, less pain, and shorter post-operative hospital stays, while achieving comparable oncologic outcomes [12,13,14]. No differences have been reported between both approaches for long-term oncologic outcomes such as 3- and 5-year survival [12]. Despite the established benefits, the uptake of laparoscopic surgery in Canada is variable and could be improved [12,13,14,15].

When examining laparoscopic surgery for colon cancer, Manitoba appears to be lagging behind. Approximately 60% of colon cancer cases are resected laparoscopically in provinces such as British Columbia and Ontario [15]. Therefore, the 24.1% of laparoscopic colon cancers in Manitoba should be viewed as a solid foundation upon which to build a stronger laparoscopic program in the province. Within Manitoba, statistically significant differences are also seen between urban and rural RHAs. When examining the two rural regions (rural 1 and 2) which have comparable resources, capacity, and equipment, the difference of 14% may be an indication of larger system or training issues. Utilizing a laparoscopic approach for rectal cancers is more complex than for colon cancers due to tumour location, and some controversies exist regarding its benefits over open resections [16]. Therefore, the lower percentage of laparoscopic rectal cancer surgery in Manitoba may be less noteworthy. Identifying these differences at the population level, and using that information to better understand the root causes, is key to supporting quality improvement efforts. Based on the findings from this study, province-wide education initiatives have been implemented to increase the use of laparoscopic surgery for colon cancers. For example, these findings prompted a needs assessment to understand the barriers to performing more laparoscopic surgery. Most surgeons indicated that additional technical skills training and the availability of a mentor to assist in cases would provide support in overcoming barriers to utilizing more laparoscopic surgery. Therefore, educational opportunities on laparoscopic surgery were offered, and a mentorship model for connecting interested surgeons with a mentor was designed. However, the mentorship opportunities were paused due to COVID-19. A notable outcome of this work is the formation of a “community of practice” among general surgeons who became further engaged in quality improvement initiatives, the impact of which will continue beyond this work. The local community of practice will act as a hub for fostering learning and sharing knowledge with the common goal of improving patient care.

Timely access to care is a cornerstone of patient-centred care, and discussions regarding quality must be accompanied by discussions regarding timeliness [17,18]. While the ultimate goal is to provide timely and high-quality surgical care, shorter wait times do not always imply improved quality of care. In some scenarios, improving quality can lead to longer wait times, and measuring quality and timeliness in conjunction provides health systems with valuable information [18]. The median number of days between first colonoscopy and first resection in our population was 40 days. A shorter median wait time was reported in the United Kingdom of 30 days between diagnosis to surgery [19]. In Manitoba, the number of days decreased with advancing stage, suggesting that the sickest patients experienced the shortest wait time. This finding has been reported in previous studies [20,21] and likely represents selection—specifically, that practitioners were able to select and more urgently treat those with more advanced disease. Individuals living in a higher income rural area had a median wait time of 41 days compared to the lowest income quintile which was 35 days. Individuals from lower socioeconomic status often present with higher stage cancer compared to those from higher socioeconomic status [22]. This observation, in combination with stage, could explain the shorter wait times among those living in lower income areas. Geographical differences are also apparent. Individuals living in rural 1 region experienced a 28-day median wait time compared to 50 days in rural 3 region. This finding aligns with geographical variations in wait times identified elsewhere within Canada [23,24].

The number of lymph nodes removed and examined in colon cancer surgery is an important quality indicator. The clinical practice guideline for lymph node removal states that at least 12 lymph nodes should be examined [10]. A higher number of lymph node removal is associated with optimal staging, lower rates of recurrence, more appropriate use of adjuvant chemotherapy, and improved survival [25]. The national target for this indicator is 90% [26]; Manitoba reported a rate of 86.1% on a population level. The lymphadenectomy ≥12 lymph nodes rate would be even higher if stage IV disease were excluded (as a proportion of these would have been for palliative intent). The target was met for stages II and III (90.9% and 89.9%, respectively) for colon cancers. While slightly lower than the national target, the lymph node removal rate in Manitoba was higher than the rates reported in some other Canadian provinces (72.6% in British Columbia; 71.4% in Nova Scotia) [26].

Circumferential resection margin (CRM) is considered a quality indicator and a strong prognostic factor in rectal cancers [11,27]. It plays a significant role in local recurrence, distant metastasis, and survival [28]. In Manitoba, 96.9% of rectal cancers had a negative CRM (i.e., 3.1% positive). A study that measured this indicator in all Canadian provinces reported a range between 7.7% to 21.1% for positive CRM [29], and other countries have reported rates up to 28% [28,30]. While surgical technique is a key factor in negative CRM rates, other important factors, including patient factors, preoperative imaging, and preoperative therapy also influence this outcome measure.

A strength of this study is the use of population-based administrative data which has been evaluated for completeness, reliability, and accuracy [31]. These data allowed for population-level geographic comparisons and contained limited missing data. A limitation of this work is the use of an area-level measure of income as a proxy for individual-level income, which may result in some misclassification of an individual’s actual income. However, several studies have reported that a correlation between area-level and self-reported individual-level income is present, and that it is a valid proxy measure [32,33]. The results from this study have laid the foundation for ongoing quality measurement in Manitoba. We will continue periodic assessments of CRC surgery and initiate quality improvement efforts as needed.

## 5. Conclusions

This study showed that high-quality colorectal cancer surgery is being conducted in Manitoba. Some variation exists between regional health authorities, income quintile, and stage, which align with the current literature documenting surgical variation. Measuring key quality indicators and establishing the infrastructure to continuously monitor progress is critical to providing high-quality care to patients. Once areas for improvement are identified, appropriate quality improvement efforts must be developed to understand the root causes of variation and to address any training or resource needs. The results from this study will be used to develop quality improvement initiatives. Moreover, we have created the program algorithms to be able to collect and analyze these population-based cancer surgery quality measures in a timely fashion and on-going basis, in order to optimize review and feedback. This is no small feat on a population level. It is important to continuously move from measurement to addressing gaps and then back to measurement to evaluate success. This will promote a culture of safety and quality improvement and will have short- and long-term benefits for patients and the health system.

## Figures and Tables

**Table 1:** Characteristics of individuals diagnosed with colon or rectal cancer, Manitoba, 2010–2014.

Characteristic	Colon n (%)	Rectal n (%)
Manitoba	2459	986
Age Group		
20–39	31 (1.3)	20 (2.0)
40–49	124 (5.0)	79 (8.0)
50–59	339 (13.8)	214 (21.7)
60–69	608 (24.7)	278 (28.2)
70–79	725 (29.5)	243 (24.6)
80+	632 (25.7)	152 (15.4)
Sex		
Male	1264 (51.4)	629 (63.8)
Female	1195 (48.6)	357 (36.2)
Income Quintile		
Urban 1 (lowest)	308 (12.8)	104 (10.8)
U2	281 (11.7)	136 (14.1)
U3	300 (12.5)	107 (11.1)
U4	255 (10.6)	103 (10.7)
U5 (highest)	272 (11.3)	121 (12.6)
Rural 1 (lowest)	182 (7.6)	88 (9.1)
R2	206 (8.6)	87 (9.0)
R3	232 (9.7)	81 (8.4)
R4	189 (7.9)	71 (7.4)
R5 (highest)	176 (7.3)	64 (6.7)
RHA of Residence (at diagnosis)		
Urban	1344 (54.7)	544 (55.3)
Rural 1	428 (17.4)	151 (15.3)
Rural 2	307 (12.5)	144 (14.6)
Rural 3	286 (11.6)	110 (11.2)
Rural 4	90 (3.7)	35 (3.6)
Stage		
Stage I	543 (22.3)	239 (24.8)
Stage II	821 (33.8)	178 (18.5)
Stage III	750 (30.8)	415 (43.0)
Stage IV	318 (13.1)	132 (13.7)
Site of Tumour		
Left Colon	1069 (44.1)	n/a
Right Colon	1353 (55.9)	n/a

**Table 2:** Surgical Approach by Regional Health Authority of Surgery for Colon Cancers, 2010–2015.

RHA of Surgery	Colon			
	Open		Laparoscopic	
	n	% (95% CI)	n	% (95% CI)
Manitoba	1631	75.8	519	24.1
Urban	1056	73.8 (71.6–76.1)	374	26.1 (23.9–28.4)
Rural 1	308	84.6 (81.2–88.5)	55	15.1 (11.5–18.8)
Rural 2	133	70.7 (64.2–77.2)	55	29.2 (22.8–35.8)
Rural 3	85	74.5 (66.6–82.6)	29	25.4 (17.4–33.4)
Rural 4	27	87.0 (75.3–98.9)	*	12.9 (1.1–24.7)

* Suppressed due to cell sizes n < 5.

**Table 3:** Surgical Approach by Regional Health Authority of Surgery for Rectal Cancers, 2010–2015.

RHA of Surgery	Rectal					
	Orifice		Open		Laparoscopic	
	n	% (95% CI)	n	% (95% CI)	n	% (95% CI)
Manitoba	61	9.2	509	77.1	90	13.6
Urban	56	11.1 (8.4–13.9)	380	75.3 (71.6–79.2)	68	13.4 (10.5–16.5)
Rural 1	*	6.0 (0.9–11.1)	67	80.7 (72.2–89.2)	11	13.2 (6.0–20.5)
Rural 2	0	0	27	81.8 (68.7–95.0)	6	18.1 (5.0–31.3)
Rural 3	0	0	23	85.1 (71.8–98.6)	*	14.8 (1.4–28.2)
Rural 4	0	0	6	85.7 (59.8–111.6)	*	14.2 (0.0–40.2)

* Suppressed due to cell sizes n < 5.

**Table 4:** Number of Days between First Colonoscopy and First Resection Among Colon Cancer Patients, 2010–2015.

Characteristic	Median (Days)	90th Percentile (Days)
Manitoba	40	119
Income Quintile		
Urban 1 (lowest)	41	122
U2	39	119
U3	43	96
U4	39	112
U5 (highest)	45	145
Rural 1 (lowest)	35	117
R2	32	114
R3	33	126
R4	48	108
R5 (highest)	41	95
RHA of Residence (at diagnosis)		
Urban	44	127
Rural 1	28	89
Rural 2	33	106
Rural 3	50	134
Rural 4	49	129
Stage		
Stage I	60	167
Stage II	35	98
Stage III	37	97
Stage IV	23	81

**Table 5:** ≥12 Lymph Nodes Removed and Negative Circumferential Resection Margin by Regional Health Authority of Diagnosis and Stage, 2010–2015.

Characteristic	≥12 Lymph Nodes Removed		Negative Circumferential Resection Margin	
	Colon Only		Rectal Only	
	n	% (95% CI)	n	% (95% CI)
Manitoba	1841	86.1	433	96.9
RHA of residence at diagnosis				
Urban	1003	86.1 (84.1–88.1)	239	96.0 (93.5–98.5)
Rural 1	313	85.8 (82.2–89.3)	68	97.1 (93.2–100.0)
Rural 2	239	87.2 (83.3–91.2)	70	98.6 (95.9–100.0)
Rural 3	217	85.1 (80.7–89.5)	42	97.7 (93.2–100.0)
Rural 4	65	85.5 (77.6–93.4)	14	100.0 (100.0–100.0)
Stage				
Stage I	308	74.8 (70.6–79.0)	106	99.1 (97.2–100.0)
Stage II	716	90.9 (88.9–92.9)	103	95.4 (91.4–99.3)
Stage III	634	89.9 (87.7–92.2)	224	96.6 (94.2–98.9)
Stage IV	182	79.5 (74.2–84.7)	n/a	

## Data Availability

Restrictions apply to the availability of these data. Data was obtained from CancerCare Manitoba Health and Seniors Care. These data are available with the permission of CancerCare Manitoba and Manitoba Health and Seniors Care.
